# An evaluation based on explainable machine learning: analysis of risk factors for enteral nutrition-related diarrhea in patients with severe stroke

**DOI:** 10.3389/fnut.2026.1893280

**Published:** 2026-08-14

**Authors:** Caiyue Xu, Wei Hu, Yuhong Wang, Di Xu, Fengzhi Chai, Yunfan Ji, Xia Li

**Affiliations:** 1 School of Nursing, Jinzhou Medical University, Jinzhou, Liaoning, China; 2 Department of Nursing, People's Hospital of Shangrao, Shangrao, Jiangxi, China; 3 First Affiliated Hospital of Jinzhou Medical University, Jinzhou, Liaoning, China

**Keywords:** enteral nutrition-associated diarrhea, interpretable models, machine learning, risk factors, severe stroke, SHAP

## Abstract

**Objective:**

This study systematically examines the clinical factors associated with enteral nutrition-related diarrhea (ENAD) in patients with severe stroke and uses interpretable machine learning methods to assess the strength of these associations.

**Methods:**

This retrospective study included 766 patients with severe stroke from two hospitals between January 2021 and December 2025; data were sourced from electronic medical records and nursing notes. The study compared eight machine learning algorithms: logistic regression (Logistic), support vector machines (SVM), random forests (RF), extreme gradient boosting (XGBoost), lightweight gradient boosting (LightGBM), classification gradient boosting (CatBoost), k-nearest neighbors (KNN), and decision trees (DT); model performance was evaluated using 10-fold cross-validation (CV); The optimal model was selected, with SHAP (Shapley Additive exPlanations) analysis used to provide feature importance and the Delong test for feature selection; the final model was evaluated and validated using metrics from calibration curves, decision curve analysis curves, and the model confusion matrix; SHAP analysis was used to interpret the final model.

**Results:**

A total of 292 patients (38.1%) in the study cohort developed diarrhea. The random forest model was identified as the optimal model and served as the feature selection model. Following the Delong test, 10 variables most closely associated with ENAD were ultimately included: duration of mechanical ventilation, number of days on enteral nutrition (EN) and formula type, C-reactive protein, white blood cell count, hemoglobin, blood urea nitrogen, room temperature, prokinetic agents, and oral potassium supplements. The model performed as follows in terms of retrospective discriminatory power: The AUC was 0.873 (95% CI: 0.826–0.920), with an accuracy of 0.836 (0.790–0.882) and a precision of 0.787 (0.697–0.875). A recall of 0.720 (0.622–0.819), an F1 score of 0.752 (0.672–0.825), and a negative predictive value of 0.859 (0.805–0.912). SHAP analysis revealed a non-linear relationship between the aforementioned features and the risk of ENAD. It should be noted that because some variables (such as duration of mechanical ventilation and number of days on enteral nutrition) reflect cumulative exposure over the entire ICU stay, reported metrics such as AUC may be overestimated and should not be directly interpreted as indicative of predictive performance. The SHAP analysis revealed patterns of association between these characteristics and the risk of ENAD.

**Conclusion:**

Based on interpretable machine learning methods, this study identified 10 factors—including duration of mechanical ventilation, number of days on enteral nutrition, and C-reactive protein levels—that were most strongly associated with ENAD in the model output in patients with severe stroke. The results suggest that, in addition to focusing on the patients' clinical condition and nutritional interventions themselves, clinicians should also pay attention to the regulation of ward temperature and the proper management of relevant medications. The clinical predictive value of these associations requires further validation through prospective, time-dependent studies.

## Introduction

As one of the leading causes of disability and death worldwide, the burden of stroke continues to rise, making it a global public health issue that demands urgent attention (1). Data from the Global Burden of Disease Study 2021 indicate that there were 11.9 million new cases of stroke worldwide in 2021 (2), with stroke-related deaths accounting for 10.7% of all global deaths (3). For patients with severe stroke, EN is a critical nutritional support measure for maintaining nutritional status and improving clinical outcomes (4–6). However, enteral ENAD, a common complication of enteral nutrition, not only undermines the effectiveness of nutritional support but also further increases the clinical and diagnostic burden on patients (7, 8). Therefore, there is a need to develop an ENAD risk prediction model for patients with severe stroke to enable early identification and precise intervention.

Diarrhea is relatively common among patients undergoing EN therapy, with an incidence rate of 12.9–44% among patients admitted to the intensive care unit (ICU) (9, 10). Guidelines and clinical evidence-based medicine regarding stroke-related EN indicate that various complications, particularly enteral nutrition intolerance, remain relatively common in clinical practice following EN support in stroke patients (11, 12). In particular, ENAD not only leads to impaired nutrient absorption and fluid and electrolyte imbalances, but also increases the risk of aspiration pneumonia, thereby prolonging the patient's hospital stay and significantly affecting the prognosis (13). Compared to general ICU patients, patients with severe stroke exhibit unique pathophysiological characteristics. Dysfunction of the brain-gut axis following stroke is the core mechanism underlying intestinal dysfunction. Cranial injury can activate the sympathetic nervous system and induce a systemic inflammatory response, leading to dysbiosis of the gut microbiota and damage to the intestinal mucosal barrier (14, 15). Furthermore, medications commonly used in the clinical management of severe stroke, such as hypertonic diuretics and broad-spectrum antibiotics, can further disrupt the intestinal microecological homeostasis, exacerbate abnormal intestinal motility, and significantly increase the risk of ENAD (16, 17). These specific factors suggest that ENAD risk assessment tools developed based on the general ICU population may have difficulty accurately assessing ENAD risk in stroke patients and thus have certain limitations in their applicability.

Currently, most predictive studies on ENAD or enteral nutrition intolerance are based on models developed from general ICU populations and rely primarily on traditional logistic regression methods; however, their predictive performance is typically limited, with the area under the curve in validation sets generally ranging from 0.69 to 0.76—a moderate level (8, 18). Furthermore, these models rarely account for the pathophysiological characteristics specific to stroke patients, which limits their practical value in the stroke population. Traditional logistic models rely on strict linear assumptions, making it difficult to accurately capture the complex non-linear relationships between patients' multidimensional clinical characteristics and complications (19). This limits their application in clinical risk stratification. With the advancement of artificial intelligence technology, machine learning models have demonstrated significant advantages in handling high-dimensional data and modeling complex relationships, and have already outperformed traditional methods in assessing risk factors for enteral nutrition in the ICU and stroke complications (20, 21). Additionally, the introduction of the SHAP method to interpret models helps address the issue of insufficient interpretability in machine learning models, thereby enhancing model transparency and improving interpretability in clinical applications to a certain extent (22, 23).

However, current systematic assessments of ENAD-related risk factors in patients with severe stroke remain limited, and there is a lack of studies utilizing interpretable machine learning methods to conduct multidimensional association analyses in this population. Accordingly, this study draws on multicenter retrospective clinical data and employs various machine learning algorithms to systematically evaluate the clinical factors associated with the onset of ENAD and the strength of these associations. The aim is to identify candidate variables for the subsequent development of a truly prospective predictive model and to provide insights for the clinical identification of high-risk individuals and the optimization of nutritional support strategies.

## Methods

### Data sources and study population

**Figure 1:**
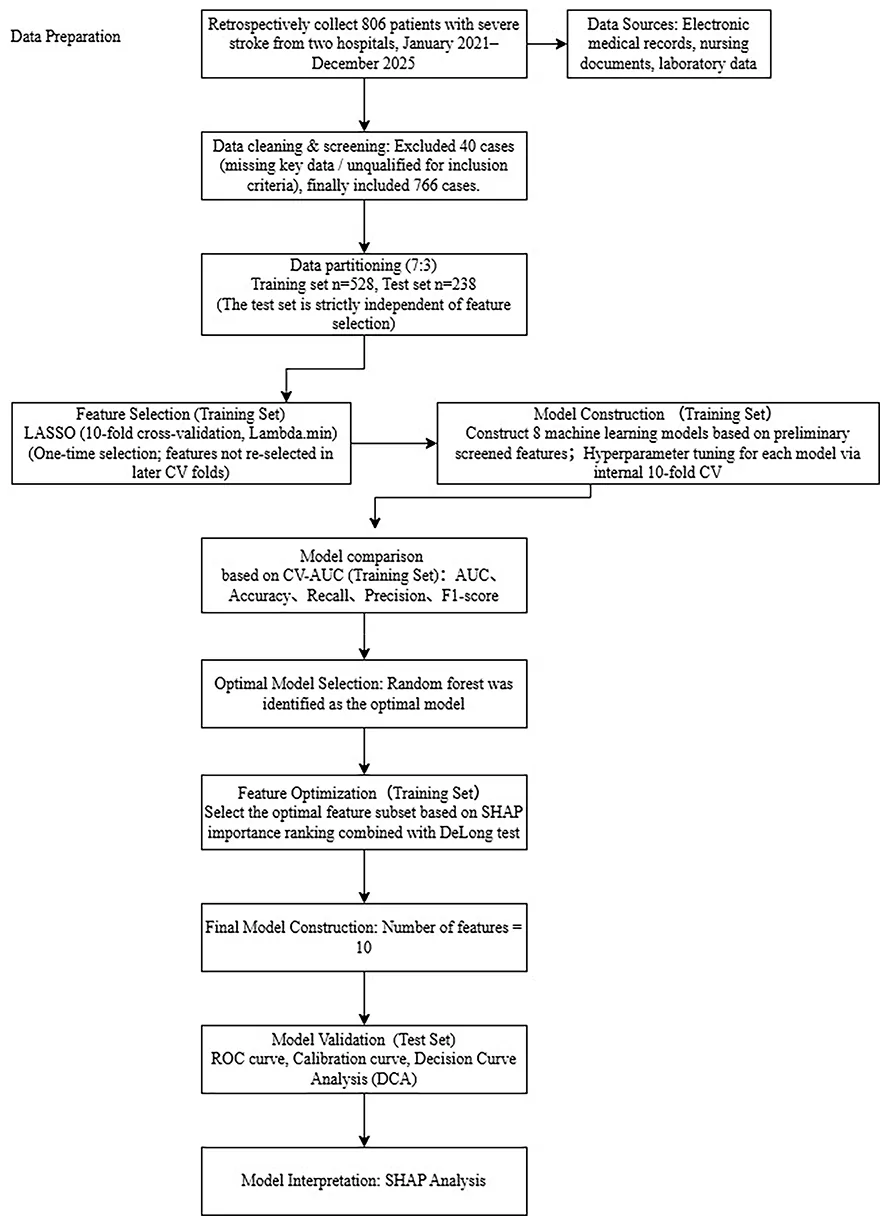
Technical roadmap for the assessment and analysis of ENAD risk factors in patients with severe stroke: the research process covers patient enrollment, data preprocessing, feature selection using the LASSO algorithm, analysis and validation, and the identification of associated factors using the SHAP algorithm. LASSO, Least Absolute Shrinkage and Selection Operator; AUC, Area Under the Receiver Operating Characteristic Curve; SHAP, SHapley Additive Explanation.

This was a multicenter retrospective cohort study that consecutively enrolled patients with severe stroke admitted to the ICU at the First Affiliated Hospital of Jinzhou Medical University, the ICU at Shangrao People's Hospital, and the Neurocritical Care Unit (NSICU) between January 2021 and December 2025. Study data were obtained from hospital electronic medical record systems, nursing records, and laboratory information systems, with clinical data extracted through a standardized process. Enrolled patients had to meet the following inclusion criteria: (1) age ≥ 18 years; (2) diagnosis of acute ischemic or hemorrhagic stroke confirmed by imaging studies such as cranial CT or MRI; (3) admission to an intensive care unit or neuro-intensive care unit with a Glasgow Coma Scale score ≤ 8; (4) APACHE II score ≥ 15, and receiving mechanical ventilation within 24 h of admission; (5) Meeting ESPEN guidelines and receiving standardized EN for ≥ 48 h. Exclusion criteria: (1) History of chronic gastrointestinal disease or gastrointestinal surgery within the past 30 days; (2) Interruption of EN for ≥24 h or missing key variables; (3) End-stage disease or expected survival time < 72 h; (4) Concurrent participation in other clinical trials related to nutritional interventions; (5) Concurrent conditions significantly affecting intestinal function, such as acute intestinal obstruction, active gastrointestinal bleeding, severe ascites, or multiple organ failure. Detailed information on the study design is shown in Figure 1. It should be noted that this study is designed as a retrospective association analysis, rather than the development of an early predictive model. The clinical variables included reflect patients' exposure throughout their entire ICU stay; for some variables (such as total duration of mechanical ventilation and total number of days on enteral nutrition), the final values may have been recorded after the onset of ENAD. Therefore, the analysis in this study aims to identify clinical factors globally associated with the occurrence of ENAD and the strength of these associations; the reported discriminatory indices, such as AUC, may be overestimated and should not be directly interpreted as indicative of prospective predictive performance.

Candidate variables were selected based on previous ENAD-related literature, ESPEN guidelines, and clinical *a priori* knowledge. Taking into account the clinical availability of ICU data, the variables were screened and consolidated; ultimately, relevant indicators routinely collected during EN treatment were included for subsequent analysis. This study determined the sample size by considering both the complexity of the model and the volume of clinically available data; the sample size design was based on the classic Event-to-Variable Ratio (EPV) criterion established by Peduzzi et al. (24). This study included a total of 292 outcome events. Initially, 35 variables were included in the modeling process, yielding an EPV of 8.3. This stage was used solely for Least Absolute Shrinkage and Selection Operator (LASSO) regression variable selection and did not involve formal modeling; after LASSO regularization, 13 variables remained, and the EPV increased to 22.5; After screening, 10 variables were ultimately included in the analysis, with an EPV of 29.2. All categorical variables in this study were entered directly in their raw form without dummy variable expansion; EPV values were calculated based on the actual number of features within the model. The data indicate that, following variable selection, the number of model variables was well-matched to the number of outcome events. Given that the large number of candidate variables in the early stages and the relatively low EPV could easily lead to overfitting, this study employed LASSO regularization for feature reduction and combined it with cross-validation for hyperparameter tuning. The entire process strictly adhered to the validation guidelines for limited-sample machine learning proposed by Vabalas et al. (25), distinguishing between feature selection, parameter optimization, and model evaluation datasets to minimize the impact of validation bias on the model's generalization performance.

### Definition of diarrhea

The study followed the definitions in the 2016 guidelines of the Society of Critical Care Medicine and the American Society for Parenteral and Enteral Nutrition (SCCM/ASPEN) (26). Patients must meet both of the following criteria to be diagnosed with ENAD: (1) Abnormal stool consistency: According to the Bristol Stool Form Scale (BSFS) (27), stool consistency is assessed as liquid or watery stools (type ≥6); (2) Altered bowel frequency or volume: ≥3 bowel movements per day, or total stool weight exceeding 500 g over 24 h. During the outcome assessment process, electronic medical record data were used to identify potential factors for non-EN-related diarrhea, including infectious diarrhea (Clostridium difficile infection, based on stool test results or confirmed clinical diagnosis records), laxative use, and other clearly documented factors that may affect bowel function (use of relevant medications or gastrointestinal diseases). Cases of non-EN-related diarrhea supported by clear evidence (positive laboratory test results or explicit medication records with temporal correlation) were excluded from the ENAD outcome assessment but were retained in the overall study population for the description of baseline characteristics and other analyses to avoid selection bias. To minimize misclassification bias as much as possible, this study prioritized objective test results and clear medication records when determining outcomes, with two investigators independently verifying the relevant information, in the event of a discrepancy, consensus was reached through discussion. The aforementioned non-EN-related factors were not included as covariates in the statistical models but were excluded during the outcome definition phase to minimize interference with the attribution of ENAD.

### Data quality control and missing value handling

This study initially collected data from 806 patients and included a total of 35 candidate variables. During the data preprocessing stage, the complete-case analysis (CCA) method was adopted, meaning only patients with complete information on key variables were retained for subsequent analysis. Cases with missing data were excluded in accordance with the principle of completeness. Ultimately, 40 patients with missing data for key variables were excluded, leaving 766 complete cases for further analysis. For continuous variables, we checked for reasonable ranges and identified outliers; outliers were corrected or removed following clinical review. For time-related variables, such as the duration of enteral nutrition and diarrhea records, we further verified the completeness and chronological order of the records to ensure the accuracy of key clinical information.

### Data collection and processing

The study employed a continuous sampling method and included a total of 766 patients with severe stroke. Seven categories of candidate variables were collected based on a predefined variable framework. The included variables comprised: demographic characteristics (age, sex, marital status); medical history and comorbidities (history of gastrointestinal disease, history of digestive system surgery, diabetes, hypertension); disease severity and clinical characteristics (diagnosis category, APACHE II, NIHSS, GCS, RASS, NRS 2002 scores, as well as mechanical ventilation and ICU length of stay);therapeutic interventions (use of PPIs, oral potassium, probiotics, sedatives, antibiotics, opioids, and gastrointestinal motility agents), laboratory parameters (white blood cell count, hemoglobin, albumin, blood urea nitrogen, calcitonin, C-reactive protein, serum sodium, serum potassium), EN-related parameters (formula, tube type, feeding method, and duration), and environmental factors (ward temperature). During the data processing phase, the study used diarrhea as the outcome variable. The dataset was divided into a training set (*n* = 528) and a test set (*n* = 238) using random sampling in a 7:3 ratio. To prevent data leakage, the test set was completely isolated during feature selection and model training.

### Statistical analysis

Statistical analysis was performed using R version 4.5.2. Continuous variables are presented as the median (interquartile range, IQR), and comparisons between groups were performed using the Mann–Whitney U test; categorical variables are presented as frequency (percentage), and comparisons between groups were performed using the Pearson χ^2^ test. Variable selection was performed using LASSO method for dimensionality reduction and feature selection. All statistical tests were two-sided; a *P*-value of < 0.05 was considered statistically significant.

### Machine learning modeling

On the training dataset, the study used LASSO regression for feature selection and developed eight machine learning algorithms for modeling and analysis, including: Logistic, SVM, RF, DT, KNN, XGBoost, LightGBM, and CatBoost. Optimal parameters for each model were selected through grid search using 10-fold cross-validation on the training set. On the independent test set, the models were comprehensively evaluated using the Area Under the Receiver Operating Characteristic Curve (AUC), Accuracy, Recall, Precision, Negative Predictive Value (NPV), and F1-score, and performed an independent evaluation of the algorithm that performed best among them. Based on the optimal model, feature selection was performed using SHAP analysis and the Delong test. Features were progressively removed in order of increasing SHAP importance, and after each removal, the fluctuations in the pruned model's CV AUC were monitored using the Delong test. The removal process was terminated when a significant decrease in CV AUC (P < 0.05) occurred, and the optimal feature set was selected based on an analysis of CV AUC stability. Fit the final analytical model using the optimal combination of features.

To ensure standardized reporting of model performance, this study evaluated models across three dimensions: discriminatory power, classification performance, and calibration. Discriminatory power was assessed using the area under the receiver operating characteristic (ROC) curve. Classification performance metrics included accuracy, recall, precision, negative predictive value, and F1 score, all reported with 95% confidence intervals. Calibration was assessed using calibration curves, calibration intercept, calibration slope, and the Brier score. All metrics were calculated based on a uniform classification threshold. Additionally, to enhance the transparency and reproducibility of the model-building process, the following methodological details are provided: hyperparameter tuning was optimized using grid search based on the training set combined with 10-fold cross-validation; no additional resampling methods were employed to address class imbalance during model training. Regarding data preprocessing, continuous variables were retained in their original form without uniform standardization or normalization; categorical variables were included in the model analysis as factors without additional dummy variable expansion. To ensure the reproducibility of the results, a fixed random seed (seed = 123) was set during model training. The model classification threshold was uniformly set to 0.5. The hyperparameter search spaces and optimal parameter settings for each model are detailed in Supplementary Materials S1 and S2.

### Ethical statement

The study protocol was approved by the Ethics Committees of the First Affiliated Hospital of Jinzhou Medical University and Shangrao People's Hospital (Ethics Approval Numbers: KYLL202543, 2024 Yiyan Lunshen No. 311). The dates of ethical approval for the two centers were October 31, 2024, and June 5, 2025, respectively. The clinical data involved were generated between January 2021 and December 2025, and all study activities were conducted after ethical approval was granted by the respective centers. This study is a multicenter retrospective observational study. All data were derived from existing clinical records and did not involve any personally identifiable information; therefore, informed consent from patients was waived upon approval by the ethics committees. During the study, all data underwent de-identification and were restricted to internal use by the research team to ensure patient privacy and data security.

## Results

The study included a total of 766 patients with severe stroke, comprising 292 patients (38.1%) in the diarrhea group and 474 patients (61.9%) in the non-diarrhea group (Supplementary Table 1). Regarding demographic characteristics, there were no significant differences between the two groups in terms of age, gender, or marital status (*P* > 0.05). Regarding comorbidities in the medical history, including a history of gastrointestinal disease, gastrointestinal surgery, diabetes, and hypertension, there were also no significant differences between the two groups (*P* > 0.05). Regarding disease characteristics, there was a significant difference in the distribution of stroke types between the two groups, with a higher proportion of intracerebral hemorrhage in the diarrhea group compared to the non-diarrhea group (*P* < 0.001). Among enteral nutrition-related factors, there was a significant difference in the type of EN formula between the two groups (*P* < 0.001), while there were no significant differences in tube type or administration method (all *P* > 0.05). Regarding medication use, the proportion of patients in the diarrhea group receiving oral potassium supplements, probiotics, sedatives, opioids, prokinetic agents, and antibiotics was significantly higher than in the non-diarrhea group (*P* < 0.05), while there was no significant difference in PPI use (*P* > 0.05). Regarding disease severity, the duration of ICU stay, duration of EN, and duration of mechanical ventilation were all significantly longer in the diarrhea group (all P < 0.001), and the APACHE II score was significantly higher (*P* = 0.002). In addition, there were significant differences between the two groups in room temperature, NRS2002 score, RASS score, and hemoglobin levels (*P* < 0.05). However, there were no significant differences in GCS score, NIHSS score, and laboratory indicators (including WBC, albumin, BUN, PCT, CRP, sodium, and potassium) (*P* > 0.05).

### Feature selection

**Figure 2:**
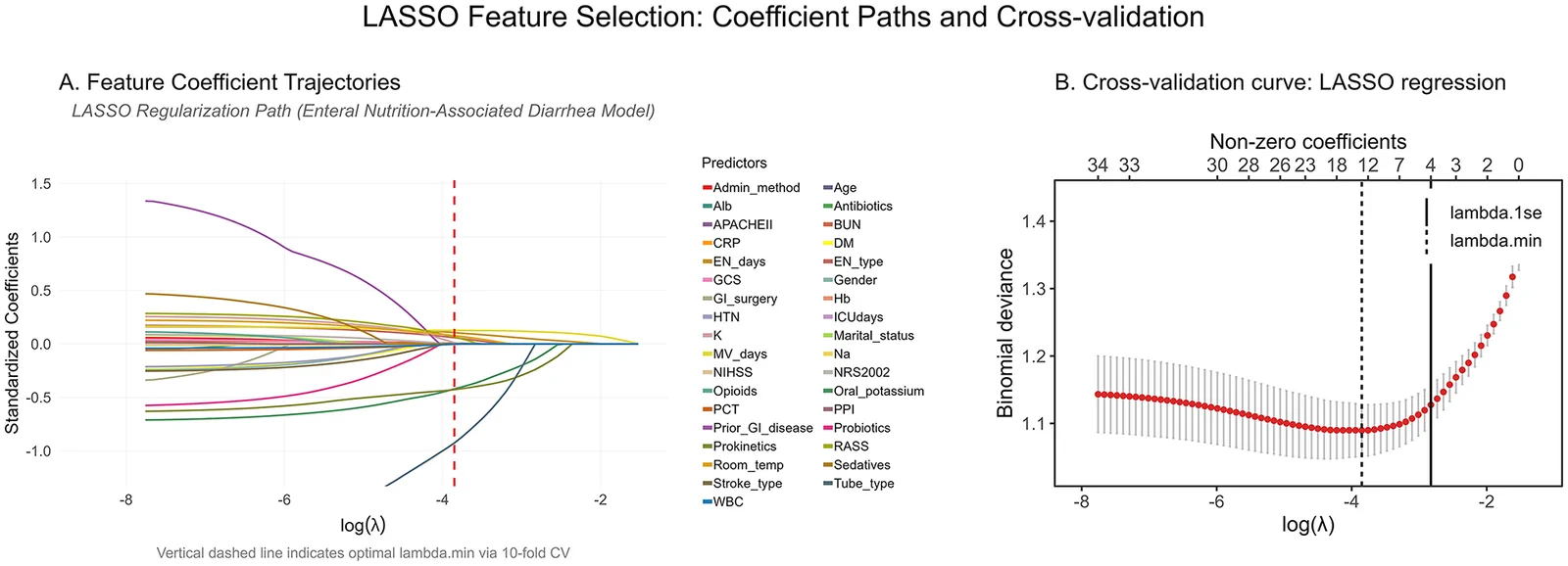
Results of LASSO regression feature selection for enteral nutrition-associated diarrhea: **(A)** The LASSO regression coefficient path plot illustrates the convergence trajectories of coefficients for each candidate variable as the penalty strength increases. Finally, the eigenvectors with nonzero coefficients at λ.min were selected for further analysis. **(B)** The optimal parameter λ was determined via 10-fold cross-validation. The figure shows the trend of the binomial bias as a function of log(λ), with the vertical dashed lines corresponding to λ.min and λ.1se, respectively.

LASSO regression analysis was performed on the training set to screen the 35 included features (Figure 2A). The optimal regularization parameter (λ = lambda.min) was determined via 10-fold cross-validation, and 13 features with non-zero coefficients were ultimately selected for subsequent analysis (Figure 2B).

### Initial model construction and performance comparison

**Figure 3:**
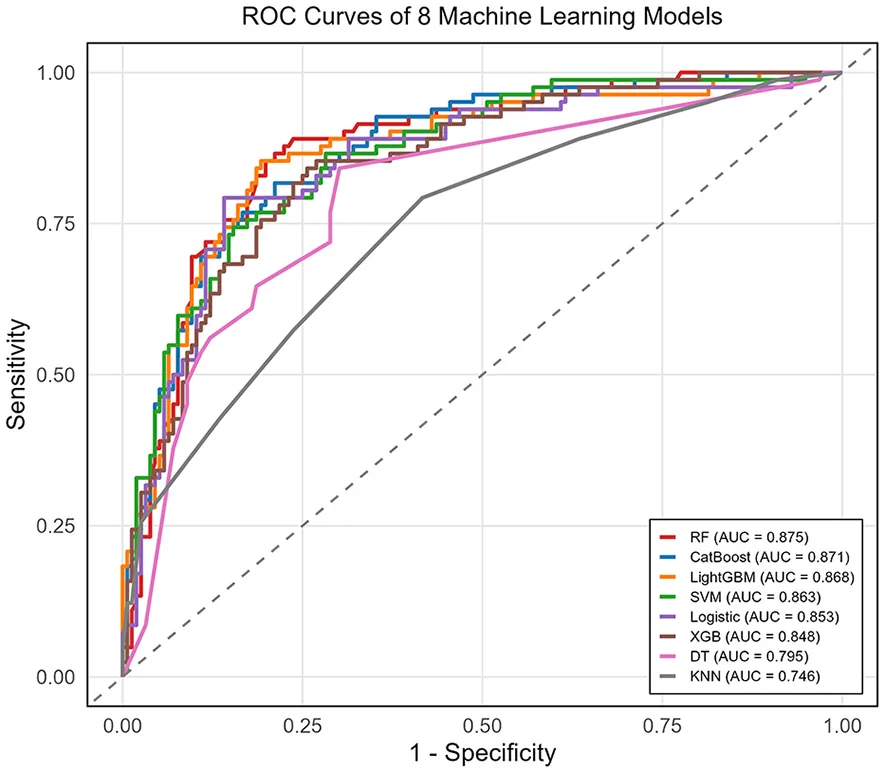
Receiver operating characteristic (ROC) curves of eight machine learning models on the independent test set: this figure shows the ROC curves for eight machine learning models in distinguishing the presence or absence of ENAD in an independent test set; the diagonal gray dashed line represents the random classification reference line.

We included the 13 features with nonzero coefficients identified by LASSO and used the training set to model and evaluate eight machine learning algorithms. The hyperparameters for each model were tuned using grid search with 10-fold cross-validation on the training set; details of the search space are provided in Supplementary File 1. A comprehensive evaluation of model performance (Table 1) revealed that RF was the optimal model. On the test set, the RF model achieved an AUC of 0.875 (95% CI: 0.829–0.921), outperforming LightGBM (0.868, 95% CI: 0.819–0.918) and XGBoost (0.848, 95% CI: 0.797–0.898). In terms of accuracy, RF (0.828, 95% CI: 0.782–0.878) performed similarly to LightGBM (0.824, 95% CI: 0.773–0.870) and ranked among the higher levels among the eight models. Although LightGBM outperformed the others in recall (0.854, 95% CI: 0.772–0.927) and F1 score (0.769, 95% CI: 0.701–0.834), its precision (0.700, 95% CI: 0.613–0.788) was lower than that of RF (0.781, 95% CI: 0.689–0.871), and RF demonstrated an advantage in reducing the risk of false positives. Given that AUC is the core metric for evaluating a model's discriminatory power, and considering that RF exhibited the most balanced and robust performance across all metrics, this study selected RF as the primary tool for subsequent association analysis. The ROC curves for each model are shown in Figure 3. It should be noted that all discriminatory and calibration performance metrics reported in this paper are calculated based on retrospective data and reflect the model's internal discriminatory ability. Since the final values for some variables (such as duration of mechanical ventilation and duration of EN) may be available after the onset of ENAD, the above metrics may be overestimated and should not be interpreted as indicative of prospective predictive performance. All subsequent performance metrics in this section are subject to this limitation and will not be addressed individually.

**Table 1:** Performance metrics (95% CI) of machine learning algorithms for assessing enteral nutrition-associated diarrhea risk in severe stroke patients.

Model	AUC (95% CI)	Accuracy (95% CI)	Recall (95% CI)	Precision (95% CI)	NPV (95% CI)	F1 score (95% CI)
Logistic	0.853 (0.801–0.905)	0.811 (0.761–0.857)	0.720 (0.623–0.817)	0.728 (0.631–0.821)	0.854 (0.797–0.906)	0.724 (0.637–0.795)
CatBoost	0.871 (0.825–0.918)	0.807 (0.756–0.857)	0.707 (0.606–0.806)	0.725 (0.630–0.821)	0.848 (0.791–0.900)	0.716 (0.632–0.788)
DT	0.795 (0.736–0.854)	0.714 (0.660–0.773)	0.720 (0.618–0.819)	0.567 (0.477–0.670)	0.828 (0.762–0.891)	0.634 (0.551–0.714)
KNN	0.746 (0.681–0.811)	0.697 (0.639–0.761)	0.573 (0.470–0.685)	0.560 (0.451–0.675)	0.773 (0.708–0.839)	0.566 (0.471–0.655)
LightGBM	0.868 (0.819–0.918)	0.824 (0.773–0.870)	0.854 (0.772–0.927)	0.700 (0.613–0.788)	0.913 (0.865–0.957)	0.769 (0.701–0.834)
RF	0.875 (0.829–0.921)	0.828 (0.782–0.878)	0.695 (0.593–0.800)	0.781 (0.689–0.871)	0.848 (0.796–0.901)	0.735 (0.654–0.813)
SVM	0.863 (0.814–0.911)	0.798 (0.748–0.849)	0.683 (0.580–0.784)	0.718 (0.615–0.819)	0.838 (0.778–0.893)	0.700 (0.610–0.778)
XGBoost	0.848 (0.797–0.898)	0.777 (0.727–0.832)	0.841 (0.757–0.916)	0.633 (0.548–0.720)	0.899 (0.848–0.947)	0.723 (0.651–0.790)

### Model feature pruning

**Figure 4:**
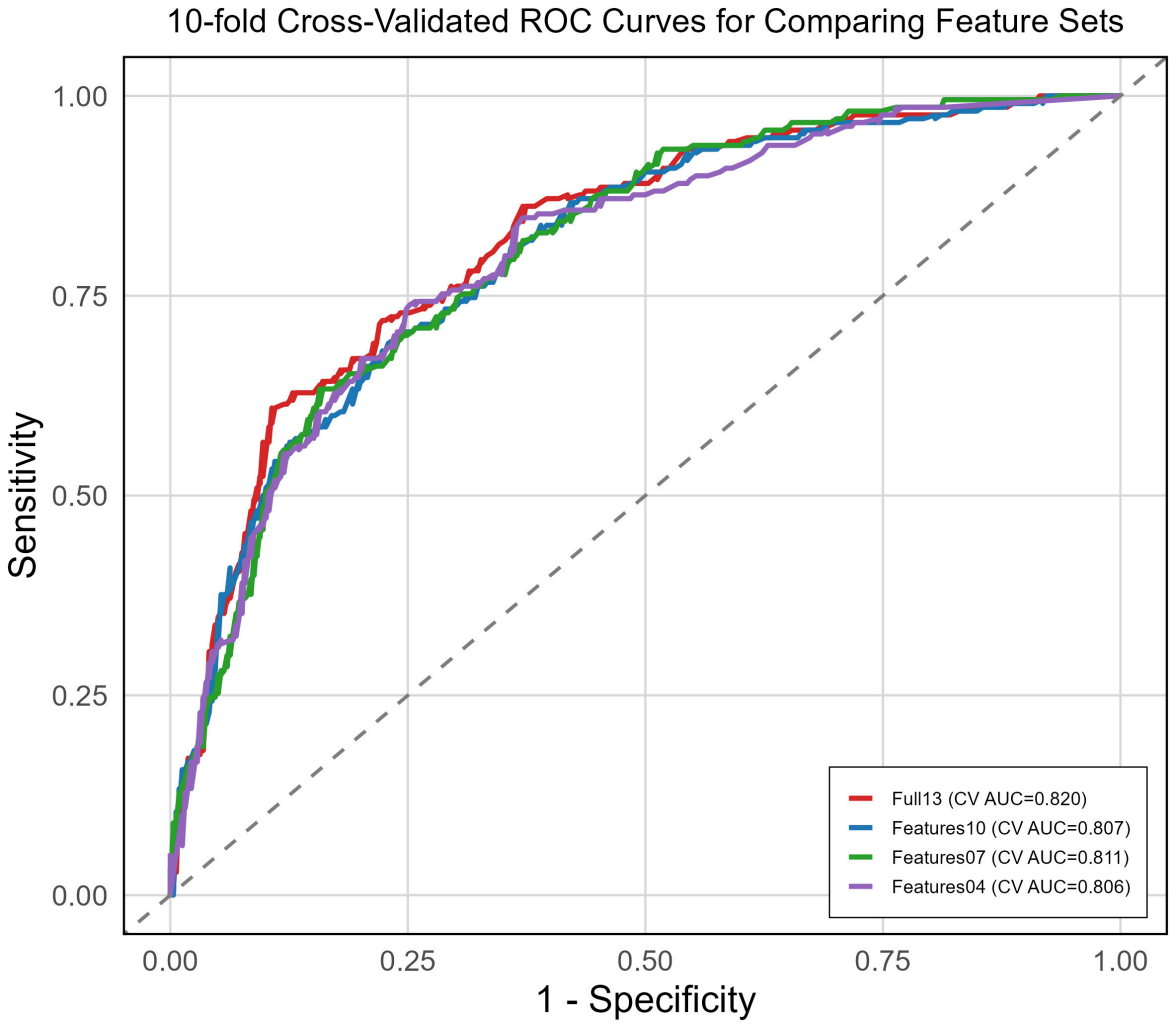
Comparison of cross-validation ROC curves for models with different feature sets: this figure shows the ROC curves and AUC values for models with four different feature sets (13, 10, 7, and 4). The AUC values represent the average area under the ROC curve obtained during 10-fold cross-validation. The diagonal gray dashed line serves as a reference line for random classification.

After RF was identified as the algorithm with the best discriminatory power in this study, feature selection was conducted to build a simplified and stable final model. It should be noted that cross-validation was used here as an internal evaluation method in the model selection process and did not result in a separate cross-validation model. On the training set, features were ranked by importance based on SHAP scores. The results showed that mechanical ventilation duration, EN duration, and the use of prokinetic agents were the primary contributing features in the model, while the contributions of the remaining features were relatively low (see Supplementary Figure S1 for details). A strategy of progressively removing low-importance features was adopted. Starting from the initial model containing 13 features, the three least important features were removed in each iteration, resulting in the construction of four pruned models with different feature combinations. To assess the stability of each pruned model, this study employed 10-fold cross-validation within the training set, and the average ROC curve and corresponding average AUC values were derived from the cross-validation results (Figure 4). Additionally, the DeLong test was used to compare the differences between each pruned model and the 13-feature model; the results are detailed in Supplementary Table S2. The results show that the differences in CV-AUC between all pruned models and the 13-feature model were not statistically significant (all *P* > 0.05), suggesting that the model's discriminative performance did not significantly decline despite the reduction in the number of features. Furthermore, to assess model stability, this study compared the widths of the 95% confidence intervals (CI) for the AUCs of each pruning model; the results are detailed in Supplementary Figure S2. The results indicate that among the pruning models, the 10-feature model had the narrowest CI width (0.163), suggesting superior stability. Based on the results regarding the discriminatory power and stability of the integrated model, a combination of 10 features was ultimately selected as the final set of variables for subsequent analysis.

### Construction and evaluation of the final analysis model

Finally, modeling and evaluation were performed using the RF algorithm based on the 10 core features identified through screening (duration of mechanical ventilation, number of days on enteral nutrition and its formulation, C-reactive protein, WBC, Hb, BUN, room temperature, prokinetic agents, and oral potassium). The model was trained on the full training set and validated on an independent test set. In terms of discriminatory ability, the final model achieved an AUC of 0.873 (95% CI 0.826–0.92) on the test set. This result was slightly lower than that of the initial model, which included 13 features (AUC = 0.875); this difference may be related to the reduced number of features. However, the differences between the two are minor, suggesting that the excluded features make only a limited contribution to the model's discriminative power. The model's performance is generally stable and demonstrates good discriminative power in the retrospective data. In terms of classification performance, the model demonstrated balanced performance across multiple evaluation metrics: accuracy of 0.836 (95% CI 0.79–0.882), recall of 0.72 (95% CI 0.622–0.819), precision of 0.787 (95% CI 0.697–0.875), NPV of 0.859 (95% CI 0.805–0.912), and an F1 score of 0.752 (95% CI 0.672–0.825). These results indicate that the model achieves a good balance in distinguishing between positive and negative outcomes.

### Explanation of the difference between CV-AUC and test set AUC

The CV-AUC values shown in Figure 4 (approximately 0.806–0.820) represent the average results from 10-fold cross-validation within the training set, reflecting the model's average stable performance across different data partitions. The AUC (0.873) reported in the main text represents the performance of the final model on a completely independent test set. Typically, because cross-validation employs a more rigorous iterative evaluation method, the CV-AUC of 0.806–0.820 is lower than the test set AUC of 0.873. The fact that the internal cross-validation estimates are lower than those of the independent test set indicates that one-time feature selection did not introduce optimism bias; the model performs robustly on independent data and does not suggest data leakage or inconsistent results. In this study, CV-AUC is used solely for feature selection and model stability assessment, while the test set AUC is used to reflect the model's actual discriminative power on an independent dataset.

### Distinguishing between different models and evaluation metrics

To avoid conceptual confusion, this study clearly distinguishes between different models and their evaluation metrics. The initial analytical model is an RF model comprising all 13 features, with an AUC of 0.875 on the test set. The pruning analytical models are candidate models constructed during the feature selection process, containing different numbers of features (10, 7, and 4, respectively). These models were used solely for cross-validation within the training set to compare the stability of different feature combinations and were not selected as the final analytical model. The cross-validation AUC represents the average performance across multiple data splits within the training set and is used to assess stability during the model selection process, rather than as a final performance metric for an independent model. The final analytical model was based on 10 core features, trained on the full training set and evaluated on an independent test set, with a test set AUC of 0.873.

### Calibration assessment and decision curve analysis

**Figure 5:**
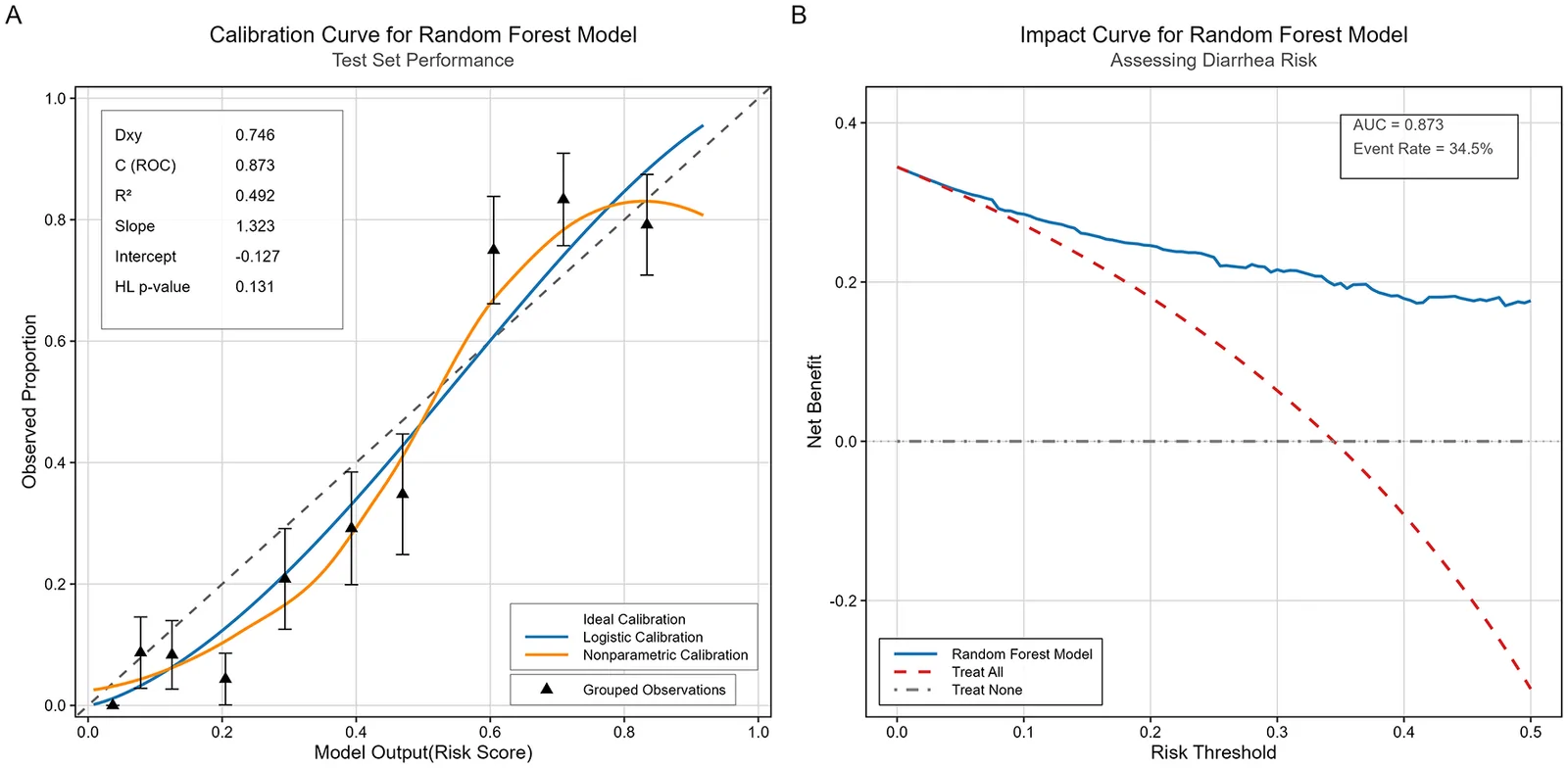
Calibration and decision curves for random forest analysis models: **(A)** Calibration curve: used to assess the consistency between the model's output risk scores and the actual observed proportions; the embedded table displays key metrics for evaluating calibration and discriminatory power; **(B)** Decision Curve Analysis (DCA): the clinical utility of the model was assessed by evaluating the clinical net benefit at different risk thresholds and comparing it with “full intervention” and “no intervention” strategies. The figure also indicates the model's AUC value and the overall event incidence rate.

All models were compared based on the same evaluation metrics, ensuring good comparability of the results. The performance of the final model in terms of discriminatory power and classification accuracy is summarized in Table 2. To further evaluate the models' calibration and clinical value, we conducted calibration curve and decision curve analyses. Results from the test set showed a Brier score of 0.138, which was lower than the Brier score (0.226) of the null model based on the positive outcome rate (34.5%), indicating that the discrimination error of this analytical model is smaller than that of the null model. The calibration slope was 1.32 and the intercept was −0.127, suggesting that calibration deviated slightly from the ideal diagonal line under a single data split (Figure 5A). To reduce the uncertainty associated with a single random split, we performed internal validation using 1,000 bootstrap resamples, evaluating calibration performance with out-of-bag samples in each resample. The results showed that the average calibration slope was 0.970 (95% CI: 0.678–1.382), the average intercept was 0.038, and the average Brier score was 0.178, indicating that the model's average calibration was close to ideal. The *P*-value for the Hosmer–Lemeshow test was >0.05; however, given that this method is sensitive to sample size and grouping, it was not considered as standalone evidence of adequate calibration. Overall, the model calibration was deemed acceptable. Regarding clinical decision value (Figure 5B), assuming a positive rate of 34.5% in the test set, the RF model demonstrated higher net benefits than both the “full intervention” and “no intervention” baseline clinical strategies across the risk threshold range of 0 to 0.5. The above results indicate that the associated factors identified in this retrospective analysis and the model's discriminatory ability demonstrate a certain degree of clinical net benefit in the retrospective data; however, this finding should not be interpreted as evidence of prospective clinical utility, and its predictive value in prospective settings still requires further validation.

**Table 2:** Performance of the final analysis model.

Metric	value	95% CI
AUC	0.873	0.826~0.92
Accuracy	0.836	0.790~0.883
Recall	0.72	0.622~0.819
Precision	0.787	0.697~0.875
NPV	0.859	0.805~0.912
F1-score	0.752	0.672~0.825
Brier Score	0.138	-
Threshold	0.509	-

### Feature correlation analysis

**Figure 6:**
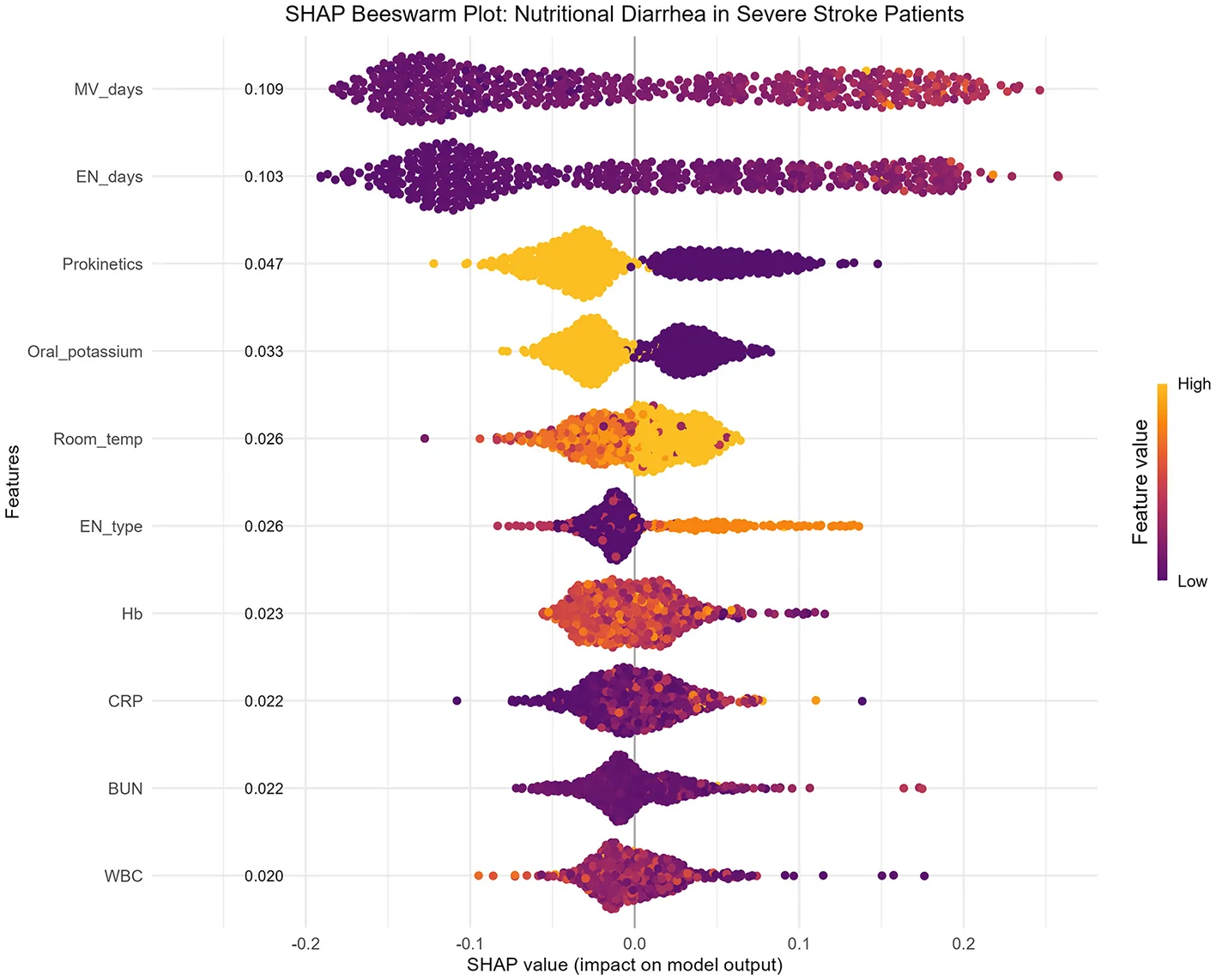
SHAP swarm plot of key features associated with ENAD in the random forest analysis model: this plot displays the top 10 most important key features and their association with the risk of developing ENAD. The position of data points on the x-axis represents SHAP values (quantifying the magnitude and direction of influence), while colors ranging from purple to yellow indicate a trend from low to high feature values, revealing the direction of the association between each feature value level and the risk of diarrhea.

**Figure 7:**
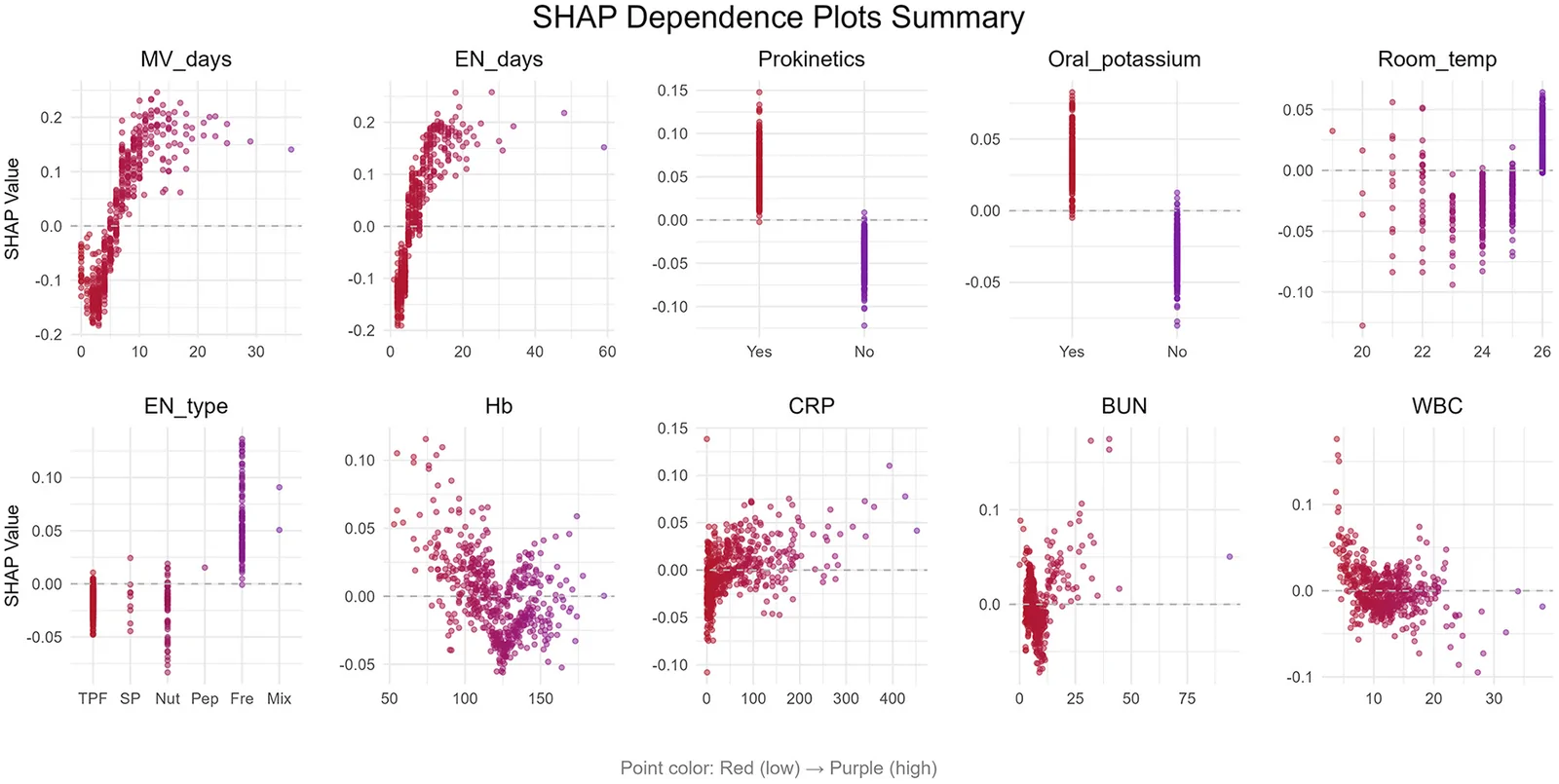
Summary of SHAP dependency graphs for individual features in the random forest analysis model: this figure illustrates the relationship between the top 10 features and their SHAP values, revealing the trends in the association between changes in these feature values and the risk of ENAD occurrence. Positive and negative SHAP values represent an association with an increased or decreased risk of ENAD, respectively; The color coding and vertical axis distribution reflect the feature value levels and their distribution characteristics within the sample.

The SHAP cluster plot (Figure 6) shows that the duration of mechanical ventilation, the duration of EN, and the use of prokinetic agents are the core characteristics most strongly associated with the risk of ENAD in patients with severe stroke, with absolute SHAP values of 0.109, 0.103, and 0.047, respectively; oral potassium supplementation (0.033), room temperature (0.026), and EN type (0.026). The SHAP dependency plot (Figure 7) further reveals the relationship between feature values and the outputs of the analytical model: as the duration of mechanical ventilation, duration of EN, room temperature, CRP, and BUN levels increase, SHAP values generally show an upward trend, suggesting that higher values of these factors are associated with an increased risk of ENAD; conversely, WBC and Hb levels show a negative correlation with the risk of ENAD, meaning that as these values increase, the corresponding risk decreases. The use of prokinetic agents and oral potassium corresponded to higher SHAP values, whereas their non-use was associated with a lower risk of ENAD. It is important to note that SHAP analysis reflects the contribution of each variable to the model's output; the results above should be interpreted as signals associated with the occurrence of ENAD, rather than as evidence of a causal relationship between the variables and the outcome.

## Discussion

This study used machine learning algorithms to systematically evaluate risk factors associated with ENAD in patients with severe stroke and compared the discriminatory capabilities of eight algorithms, among which the RF algorithm demonstrated better overall performance. A total of 766 patients with severe stroke were included, of whom 292 (38.1%) developed diarrhea meeting the criteria. Ultimately, the 10 variables most closely associated with ENAD were included in the final analytical model. Among the variables included in the model, each indicator reflects the underlying mechanisms of ENAD occurrence at different levels; the discussion is based on the performance of each feature as depicted in the single-feature dependency graph.

In terms of disease severity, prolonged mechanical ventilation is generally regarded as an indicator of worsening condition. The dependency plot shows that the risk of diarrhea among patients on mechanical ventilation exhibits a clear trend of change over time: it rises sharply in the early stages of ventilation (the first 10 days), then slows after 20 days and enters a plateau phase. This is consistent with the findings of Heyland et al. (28), who observed that gastrointestinal complications in mechanically ventilated patients peaked on days 4~5 and then gradually stabilized; Other studies have shown that diarrhea is common among mechanically ventilated patients (29). The mechanism underlying this “plateau effect” can be understood through the phasic evolution of pathophysiology in critically ill patients (5, 30). During the acute phase of ventilation, severe stress combined with high-dose sedation and broad-spectrum antibiotic use causes significant damage to the intestinal mucosa and microbiome, leading to a sharp increase in risk (31). As the duration of mechanical ventilation increases, patients gradually transition into a chronic critical illness state (32). At this stage, inflammation subsides, tracheostomy reduces the need for sedation (33), and the gut microbiota reaches a new, lower steady state under intervention (34). This observed pattern suggests the importance of prioritizing the “window of opportunity” for intervention and shifting the focus of prevention to an earlier stage. This will enable healthcare providers to implement dynamic monitoring of gastrointestinal function and intensive management during critical periods, thereby contributing to better clinical outcomes.

Regarding EN-related characteristics, both the duration of EN and the formula type exhibit relatively independent patterns of influence, and there may be some interaction between them. First, this study observed that the relationship between EN duration and the risk of diarrhea follows a “rise-then-plateau” trajectory: within the first 15 days of feeding, the risk increased with time; however, after 30 days, the risk gradually stabilized or even decreased slightly. During the acute phase of stroke, the body is in a state of sympathetic hyperactivity, which can lead to impaired intestinal mucosal barrier integrity. Furthermore, reduced gastrointestinal perfusion caused by visceral vasoconstriction, combined with insufficient secretion of digestive enzymes, results in impaired initial tolerance of the intestine to nutritional input (35). As the disease progresses into the stable phase, the remaining intestinal structure and function gradually undergo compensatory adaptation, causing the risk of diarrhea to cease its upward trend (36). Furthermore, this plateau phenomenon may also be influenced by clinical selection bias: patients who experience severe feeding intolerance early on are often downgraded or switched to parenteral nutrition, whereas patients who are able to maintain EN long-term inherently possess better gastrointestinal tolerance. Secondly, regarding formula type selection, this study indicates that high-energy-density, high-protein formulations (TP-HE) are associated with a higher risk of diarrhea (SHAP values are higher in this range). The physicochemical properties of such formulas may help explain this association: on the one hand, high-energy-density formulas typically have high osmolarity, which readily creates a hyperosmotic gradient in the intestinal lumen, promoting the reverse transport of water into the lumen and thereby triggering osmotic diarrhea (26); on the other hand, in critically ill patients with impaired intestinal digestive and absorptive capacity, unabsorbed macromolecular proteins and fats enter the colon, where abnormal fermentation by the gut microbiota produces excessive gas and metabolic byproducts, further exacerbating diarrhea symptoms (37). Overall, there appears to be a dynamic interaction between the timing of EN and the characteristics of the formula. The early stages of illness and feeding represent a period of extreme intestinal barrier fragility; during this time, the premature use of hyperosmolar, high-energy formulas may increase the risk of diarrhea. Therefore, clinicians may consider adopting a “stepwise” nutritional intervention strategy (5): prioritize isotonic formulas during the initial feeding phase, strictly controlling the initial infusion rate and concentration; Once the patient's condition has stabilized and gastrointestinal tolerance has improved, a gradual transition to high-energy-density formulations should be initiated. Concurrently, nursing staff should intensify dynamic monitoring of abdominal signs and bowel movements to promptly identify signs of intolerance, thereby ensuring adequate nutrient supply while maximizing the stability of the intestinal microbiota and function.

In terms of medication-related factors, prokinetic agents and oral potassium supplements showed high importance in the model output in this study. It should be emphasized that analysis based on the SHAP method is primarily used to explain the contributions of various variables to the model's output and cannot be used to infer causal relationships. Therefore, these variables should be understood as indicators associated with the occurrence of diarrhea, rather than definitive causative factors. Patients with severe stroke often experience delayed gastric emptying due to central nervous system damage and exposure to sedative medications, necessitating the routine use of prokinetic agents (6). Previous studies have suggested an association between the use of such medications and the incidence of diarrhea in critically ill patients (38), which is consistent with the findings of this study. Research by Nguyen et al. and recent evidence-based medical literature also indicate that the use of prokinetic agents is associated with the occurrence of noninfectious diarrhea in critically ill patients, and that diarrhea symptoms typically resolve after discontinuation of these medications (39, 40). However, this association may also reflect, to some extent, the severity of the patient's illness or the gastrointestinal motility disorder itself, rather than a direct causal effect of the medication. Oral potassium supplements have also been identified as a factor associated with the occurrence of diarrhea. Critically ill patients with dysphagia often require oral potassium supplementation, which is typically administered clinically via an EN tube in the form of liquid potassium preparations. Such preparations have a high osmolarity, typically exceeding the tolerance level of the intestinal mucosa (41). The combination of hyperosmolar potassium salts and EN solutions in the intestinal lumen can increase the osmotic load within the lumen, creating a high osmotic gradient that causes fluid to leak into the intestinal lumen. This disrupts the gastrointestinal absorption rhythm of the nutritional solution and is associated with the occurrence of osmotic watery diarrhea (42). Furthermore, some liquid potassium preparations contain non-absorbable sugar alcohols, such as sorbitol, which may ferment in the colon and exacerbate the risk of diarrhea during tube feeding (43). It is also important to note that the use of oral potassium supplements may reflect electrolyte imbalances or disease severity, which could influence the aforementioned associations. Therefore, the identification of these characteristics provides a reference for ENAD risk assessment, but they should be interpreted as associated factors rather than independent causal risk factors. In clinical practice, for EN patients experiencing diarrhea, attention should be paid to the issue of hyperosmolar load associated with enteral medications. Before administering hypertonic electrolyte solutions via the feeding tube during EN, they should be properly diluted with sterile water; for patients who have already developed severe diarrhea, potassium supplementation via the EN tube may be temporarily switched to the intravenous route—following consultation with a physician—to reduce the intestinal burden and promote intestinal mucosal repair (44). At the same time, dynamic monitoring can be conducted by assessing changes in bowel sounds and using the Bristol Stool Scale (27) to strike a balance between promoting gastric emptying and protecting intestinal absorption function.

Abnormal laboratory findings may serve as potential markers in patients with severe stroke and ENAD. This study examined two categories of markers: inflammatory markers (WBC, CRP) and physiological and biochemical markers (Hb, BUN). Previous studies have suggested that elevated WBC levels indicate infection and worsening inflammation, increasing the risk of EN intolerance (13). However, this study observed that lower white blood cell counts (< 10 × 10^9^/L) were actually associated with a higher risk of diarrhea. This discrepancy may be related to the pathophysiological state of patients with severe stroke. Severe stroke may induce stroke-induced immunosuppression syndrome (SIDS), which reduces the body's ability to fight infections (45); low WBC levels indicate that patients are in a state of frailty, which not only weakens the defensive capabilities of the intestinal mucosal barrier but also exacerbates gut microbiota dysbiosis and bacterial translocation (46). Therefore, in patients with severe stroke, low WBC levels may serve as a warning sign for intestinal dysfunction and the onset of diarrhea. Analysis of CRP levels showed that within the 0–50 mg/L range, the risk of diarrhea increased with rising CRP levels and stabilized at >100 mg/L. This observation is generally consistent with previous understanding of how systemic inflammatory responses affect gastrointestinal function (47). Elevated CRP levels reflect an increased systemic inflammatory burden. The inflammatory cascade not only causes damage to the intestinal mucosal barrier, increased intestinal permeability, and motility disorders (8), but also leads to an imbalance in the gut microbiota (48), collectively contributing to the occurrence of diarrhea.

A certain non-linear association was observed between indoor temperature and the risk of diarrhea. This study observed that the risk of diarrhea rose slightly when ward temperatures ranged from 20 to 22 °C, remained relatively stable in the 23–25 °C range, and began to rise again at approximately 26 °C. The temperature data in this study were derived from records of fixed monitoring devices in the ICU, reflecting environmental temperature at the ward level rather than real-time measurements at individual bedside locations. Furthermore, continuous dynamic monitoring and time-weighted averaging were not performed, and the temporal sequence between temperature and the occurrence of diarrhea could not be clearly established. The above results suggest that there may be a potential range of ward temperatures associated with EN intolerance; however, this association should be interpreted with caution and may be influenced by various factors, including center-to-center differences, seasonal variations, the preparation and infusion duration of enteral formulations, and nursing practices. This non-linear relationship may involve multiple contributing mechanisms. First, ambient temperature is considered one of the key factors influencing microbial proliferation. Under higher room temperature conditions, enteral nutrition solutions left hanging for extended periods may be more prone to microbial contamination, thereby potentially increasing the risk of infectious diarrhea (49). Second, environmental physical factors may indirectly interfere with gastrointestinal function by affecting the body's thermal balance and fluid status. For example, changes in room temperature may affect peripheral blood flow distribution and insensible water loss; in critically ill patients, these changes may be associated with inadequate intestinal perfusion and functional disturbances (50). Previous studies suggest that management of the ICU microenvironment (including temperature and humidity control and the duration of intravenous fluid administration) is associated with nosocomial infections and gastrointestinal complications (51). A prospective study also found that maintaining the ICU temperature within the range of 22–24 °C may help inhibit bacterial growth in nutritional solutions during prolonged infusions, which is associated with a reduced risk of infectious diarrhea (52). Therefore, indoor ambient temperature, as a modifiable factor, may have potential significance in ENAD risk management. However, as this study is a retrospective analysis, the above results only suggest a statistical association and should not be interpreted as a causal relationship; nor should they be directly interpreted as indicators of prospective clinical utility. Their independent association and potential clinical significance still require further validation in prospective studies.

### Exploratory clinical implications and future research

The associated factors identified in this retrospective case-control study may provide some guidance for the clinical management of patients with severe stroke using the ENAD scale. Compared with traditional logistic regression models, machine learning models are better able to capture the complex non-linear relationships among multidimensional clinical variables and offer greater flexibility in exploratory risk factor assessment. At the same time, the SHAP method was introduced to interpret the model's output, allowing the contributions of each variable to the model output for individual patients to be quantified and visualized, which helps clinicians understand the relative roles of each factor in risk determination. This study suggests that clinical nursing should focus on the following areas: For patients in the early stages of mechanical ventilation and at the onset of EN, monitoring of gastrointestinal function should be intensified (53); For patients who require hypertonic medications (such as oral potassium preparations) administered via enteral feeding, ensure that the medication is properly diluted or evaluate alternative routes of administration (41); During nutritional support, it is important to follow the principles of isotonic formulas and gradual feeding escalation (5, 54), and bowel movements should be dynamically monitored using the Bristol Stool Scale (27); At the same time, maintain an appropriate room temperature and implement enhanced, detailed management of nutritional solutions for patients with compromised immune function (55). In the future, if the associated factors identified in this study are validated in prospective, time-dependent studies, consideration could be given to integrating the relevant variables into electronic health record systems to develop real-time risk assessment tools that are truly applicable in clinical practice (56). However, at this stage, the findings of this study should be viewed as hypothesis generation and variable selection; they are not yet sufficient to directly guide individualized clinical decision-making.

### Limitations

This study has several limitations. First, regarding the study design and model specification, this study is a retrospective study. Some of the analytical variables are derived from dynamic clinical data collected during patients' ICU treatment, which may reflect changes in disease severity and treatment strategies rather than merely representing the patients' initial status. It is worth noting that some time-dependent variables (such as duration of EN, duration of mechanical ventilation, and drug exposure) are constructed based on cumulative information during the ICU stay and may include data collected after the outcome event occurred, thereby introducing potential temporal bias. Furthermore, this study did not truncate time-dependent variables or employ time-dependent modeling methods; Future studies could conduct prospective validation of the associated factors identified in this study using time-dependent methods—such as threshold analysis or combined models—based on a clearly defined prediction time window. Therefore, the results of this study should be interpreted as a retrospective association analysis; performance metrics such as AUC may be overestimated due to the inclusion of *post hoc* information and should not be interpreted as indicative of prospective predictive performance. Ambient temperature, as a ward-level indicator, is recorded using fixed monitoring equipment in the ICU; it has not undergone individualized or continuous dynamic assessment and may be influenced by various factors such as seasonal variations, center-specific differences, and nursing practices. As such, it exhibits characteristics of a surrogate marker, which may introduce residual confounding. This affects the generalizability of the results. Therefore, in future prospective studies, more thorough control for seasonal factors, center-specific differences, and nursing protocols could be implemented to verify their independent associations and general applicability. Second, although internal stability was assessed using 10-fold cross-validation, the study constructed the model based on data from two centers; differences between centers in EN strategies and nursing procedures may affect model performance. Additionally, the study used randomly split training and test sets for model evaluation; both the training and test sets may have included patients from different centers, failing to achieve external validation based on independent centers. This validation strategy may to some extent overestimate the model's discriminatory power. Furthermore, although 1,000 bootstrap resamples were used to evaluate the stability of the calibration curve, bootstrap-based optimism correction was not applied to the complete model development pipeline (including feature selection, hyperparameter tuning, and model fitting). Therefore, the reported performance metrics may still be subject to optimistic bias. Future research could further optimize validation strategies—such as employing nested cross-validation or applying bootstrap optimism correction to the entire modeling pipeline—to more comprehensively assess the stability and generalizability of model performance. Third, regarding data handling, this study employed a strategy of removing entire rows containing missing values. Since missing data are not entirely random, this method may reduce the effective sample size and introduce selection bias, thereby affecting the model's generalization ability and the robustness of the results. Furthermore, the clinical characteristics of excluded patients may differ systematically from those of the included population, potentially influencing the assessment of model performance. Therefore, future studies could further validate missing data handling strategies by employing multiple interpolation methods or conducting sensitivity analyses to enhance the reliability of the research findings. Fourth, regarding the assessment of outcome variables, the diagnosis of diarrhea primarily relied on clinical evaluations by nursing staff based on the Bristol Stool Form Scale. Although this method is widely used in clinical practice, it may still be subject to certain subjective factors, thereby introducing measurement bias. Furthermore, this study did not quantitatively assess inter-rater reliability, and potential inter-rater differences may exist. Future studies could further improve the reliability of assessments through standardized training and inter-rater reliability tests. In defining the outcome, although this study made every effort to identify and exclude infectious diarrhea, laxative use, and other factors unrelated to enteral nutrition through electronic health records, due to limitations in the completeness of retrospective data and the accuracy of clinical records, the cause of diarrhea in some cases may remain unclear, thereby introducing a certain degree of misclassification bias. Therefore, prospective studies incorporating more standardized diagnostic procedures are still needed to further validate the findings of this study. Fifth, there are certain limitations regarding the inclusion of variables. This study only considered the use of antibiotics without further distinguishing their type, dosage, or duration of treatment; simultaneously, some key nursing factors (such as EN infusion rate and positioning management) were not quantified and included in the model, which may have led to residual confounding. Finally, at the clinical application level, the widespread adoption of this model remains constrained by factors such as information system support and human resource allocation, limiting its scope of application. Therefore, this model is more suitable as a clinical assessment aid rather than as an independent basis for decision-making. Finally, the findings of this study are still at an exploratory stage. Even if they are validated by future prospective studies and considered for clinical translation, they will still be subject to constraints such as information system support and human resource allocation. Therefore, at this time, the findings of this study are more suitable as an exploratory reference for risk screening rather than as a basis for clinical decision-making. Future prospective studies with predefined prediction time windows are needed to further validate the model's performance, and multicenter, large-sample studies are required to optimize and externally validate the model, thereby enhancing its stability and clinical applicability.

## Conclusion

Based on multicenter clinical data—specifically, multicenter retrospective data—this study used interpretable machine learning methods to systematically evaluate risk factors associated with ENAD in patients with severe stroke. The results indicate that factors such as duration of mechanical ventilation, duration of EN, type of EN formula, use of prokinetic agents and oral potassium, CRP levels, and room temperature are the candidate factors most strongly associated with the model's output. In comparison, RF demonstrates better overall discriminatory power among the various algorithms. Combined with SHAP analysis, this study further identified key features and provided an intuitive interpretation of the model's output. From a clinical perspective, most of these factors can be obtained through routine monitoring, providing clues for identifying patients at high risk for ENAD. However, due to the retrospective design of this study and potential temporal information leakage for some variables, the findings should be interpreted as exploratory associations; their prospective predictive value requires further confirmation through independent external validation and time-dependent studies.

## Data Availability

The raw data supporting the conclusions of this article will be made available by the authors, without undue reservation.

## Supplementary material

Supplementary Figure 1 SHAP Global Importance Analysis Based on LASSO Feature Selection: the overall contribution weights of the 13 features were quantified and ranked using the average absolute SHAP values to evaluate the importance of each variable in the analysis and the strength of its association with the risk of ENAD.

Supplementary Figure 2 Model stability assessment across different feature sets: Compares the performance stability of four feature sets using 10-fold cross-validation. Performance variability is quantified by the width of the 95% confidence interval for AUC; smaller values indicate the model exhibits greater robustness in distinguishing between different subsets.
