# Supplementary material for: An evaluation based on explainable machine learning: analysis of risk factors for enteral nutrition-related diarrhea in patients with severe stroke
Source: Front Nutr. 2026 Aug 14;13:1893280. doi: 10.3389/fnut.2026.1893280 (PMC13521873; doi:10.3389/fnut.2026.1893280)
Supplement: Supplementary file 1 [file Supplementary_file_1.docx]

**Supplemental file1.**

**Hyperparameter Settings and Grid Search Ranges for Machine Learning Models**

**SVM Hyperparameter Search Range and Optimization Results**
**Hyperparameter Search Range**

Range C (regularization parameter)

Search range: {0.1, 0.5, 1.0, 2.0, 5.0, 10.0}

sigma (kernel parameter)

Search range: {0.001, 0.005, 0.01, 0.05, 0.1, 0.5, 1.0, 2.0}

kernel (kernel type)

Search range: {"radial"}

**Optimal Values**

C = 0.1

sigma = 0.05

kernel = "radial"

**Random Forest Hyperparameter Grid Search Range and Optimal Values：**

mtry (number of variables selected for each split)

Search range: {2, 3, 4, 5, 6}

**Optimal Values**

mtry = 3

**LightGBM Hyperparameter Search Range and Optimization Results**

**Hyperparameter Search Range**

num_leaves (number of leaves in one tree)

Search range: {31, 63}

learning_rate (shrinkage rate)

Search range: {0.01, 0.05, 0.10}

**Optimal Values**

num_leaves = 63

learning_rate = 0.05

**Logistic Regression Hyperparameter Search Range and Optimization Results**

**Hyperparameter Search Range**

alpha (mixing parameter, 0 = ridge, 1 = lasso)

Search range: {0.0, 0.2, 0.4, 0.6, 0.8, 1.0}

lambda (regularization parameter)

Search range: {0.0001, 0.001, 0.01, 0.05, 0.1, 0.5, 1.0, 2.0, 5.0}

**Optimal Values**

alpha = 0.0

lambda = 0.1

Ridge regression (L2 penalty)

**Decision Tree Hyperparameter Search Range and Optimization Results**

**Hyperparameter Search Range**

cp (complexity parameter)

Search range: {0.001, 0.003, 0.005, ..., 0.099}

**Optimal Values**

cp = 0.001

**XGBoost Hyperparameter Search Range and Optimization Results**

**Hyperparameter Search Range**

max_depth (maximum tree depth)

Search range: {3, 5, 7}

eta (learning rate)

Search range: {0.05, 0.10, 0.20}

gamma (minimum loss reduction)

Search range: {0, 0.1}

**Optimal Values**

max_depth = 3

eta = 0.10

gamma = 0.1

**CatBoost Hyperparameter Search Range and Optimization Results**

**Hyperparameter Search Range**
depth (tree depth)
Search range: {3, 4, 5, 6}
learning_rate (learning rate)
Search range: {0.01, 0.03, 0.05, 0.1}
l2_leaf_reg (L2 regularization coefficient)
Search range: {1, 3, 5}
iterations (number of trees)
Search range: {100, 200, 300, 500} (early stopping with od_wait=50)

**Optimal Values**
iterations = 100 (best iteration = 30, determined by 10‑fold CV early stopping)
depth = 6
learning_rate = 0.1
l2_leaf_reg = 5

**KNN Hyperparameter Search Range and Optimization Results**

**Hyperparameter Search Range**

k (number of neighbors)

Search range: 1, 3, 5, 7, 9, 11, 13, 15, 17, 19, 21

**Optimal Values**

k = 11

**10 Features Random Forest Hyperparameter Grid Search Range and Optimal Values：**

**Hyperparameter Grid Search Range**

mtry (number of variables randomly sampled as candidates at each split)

Search range: {2, 3, 4, 5, 6}

**Optimal Values**

mtry = 2
