# Supplementary material for: An evaluation based on explainable machine learning: analysis of risk factors for enteral nutrition-related diarrhea in patients with severe stroke
Source: Front Nutr. 2026 Aug 14;13:1893280. doi: 10.3389/fnut.2026.1893280 (PMC13521873; doi:10.3389/fnut.2026.1893280)
Supplement: Supplementary file 2 [file Table_1.docx]

Supplemental Table 1. Demographic and clinical characteristics of severe stroke patients by diarrhea status

| Characteristics | Subgroups | All patients  (n=766) | Diarrhea  (n=292) | Nondiarrhea  (n=474) | P value |
| --- | --- | --- | --- | --- | --- |
| Gender | Male | 473(61.7) | 181(38.3) | 292(61.7) | 0.916 |
|  | Female | 293(38.3) | 111(37.9) | 182(62.1) |  |
| Stroke type | ICH | 530(69.2) | 226(42.6) | 304(57.4) | <0.001 |
|  | CI | 236(30.8) | 66(28.0) | 170(27.4) |  |
| Marital | Single | 21(2.7) | 9(42.9) | 12(57.1) | 0.991 |
|  | Married | 678(88.5) | 258(38.1) | 420(61.9) |  |
|  | Divorced | 26(3.4) | 8(30.8) | 18(69.2) |  |
|  | Widowed | 41(5.4) | 17(41.5) | 24(58.5) |  |
| P-GIDx,n(%) | Yes | 26(3.4) | 7(26.9) | 19(73.1) | 0.232 |
|  | No | 740(96.6) | 285(38.5) | 455(61.5) |  |
| GISurg,n(%) | Yes | 30(3.9) | 9(30) | 21(70) | 0.35 |
|  | No | 736(96.1) | 283(38.5) | 453(61.5) |  |
| Diabetes,n(%) | Yes | 124(16.2) | 54(43.5) | 70(56.5) | 0.174 |
|  | No | 642(83.8) | 238(37.1) | 404(62.9) |  |
| Hypertension,n(%) | Yes | 446(58.2) | 168(37.7) | 278(62.3) | 0.761 |
|  | No | 320(41.8) | 124(38.8) | 196(61.3) |  |
| Tube type,n(%) | Gastric tube | 723(94.4) | 280(38.7) | 443(61.3) | 0.156 |
|  | Nasoenteral tube | 43(5.6) | 12(27.9) | 31(72.1) |  |
| Admin method,n(%) | CP | 607(79.1) | 232(38.3) | 375(61.8) | 0.598 |
|  | IP | 38(5.0) | 13(34.2) | 25(65.8) |  |
|  | Bolus | 121(15.8) | 46(38) | 75(62) |  |
| EN type,n(%) | TPF | 500(65.3) | 46(38.0) | 75(62) | <0.001 |
|  | SP | 15(2.0) | 4(26.1) | 11(73.3) |  |
|  | Nutison | 78(10.2) | 17(21.8) | 61(78.2) |  |
|  | Peptison | 1(0.1) | 1(100) | 0(0) |  |
|  | Fresubin | 170(22.2) | 81(47.6) | 89(52.4) |  |
|  | Mixed feeding | 2(0.3) | 2(100) | 0(0) |  |
| PPI,n(%) | Yes | 529(69.1) | 196(37.1) | 333(62.9) | 0.363 |
|  | No | 237(30.9) | 96(40.5) | 141(59.5) |  |
| Oral potassium,n(%) | Yes | 364(47.5) | 180(49.5) | 184(50.5) | <0.001 |
|  | No | 402(52.5) | 112(27.9) | 290(72.1) |  |
| Probiotics,n(%) | Yes | 52(6.8) | 30(57.7) | 22(42.3) | 0.003 |
|  | No | 714(93.2) | 262(36.7) | 452(63.3) |  |
| Sedatives,n(%) | Yes | 679(88.6) | 273(40.1) | 407(59.9) | 0.002 |
|  | No | 87(11.4) | 20(23.0) | 67(77.0) |  |
| Opioids,n(%) | Yes | 309(40.3) | 138(44.7) | 171(55.3) | 0.002 |
|  | No | 457(59.7) | 154(33.7) | 303(66.3) |  |
| Prokinetics,n(%) | Yes | 322(42.0) | 172(53.4) | 150(46.6) | <0.001 |
|  | No | 444(58.0) | 120(27.0) | 324(73.0) |  |
| Characteristics | Subgroups | All patients  (n=766) | Diarrhea  (n=292) | Nondiarrhea  (n=474) | P value |
| Antibiotics,n(%) | Yes | 698(91.1) | 276(39.5) | 422(60.5) | 0.009 |
|  | No | 68(8.9) | 16(23.5) | 52(76.5) |  |
| ICUdays (days),M(IQR) |  | 7(4-11) | 10(7-14) | 5(3-8) | <0.001 |
| Age(years),M(IQR) |  | 64(55-72) | 64(55-72) | 64(55-72) | 0.886 |
| Room temp  (℃),M(IQR) |  | 26(24-26) | 26(24-26) | 25(24-26) | <0.001 |
| EN days(days),M(IQR) |  | 5(3-9) | 8(5-12) | 4(3-6) | <0.001 |
| MVdays(days),M(IQR) |  | 5(3-9) | 8(6-11) | 3(2-6) | <0.001 |
| APACHE II,M(IQR) |  | 23(19-26) | 24(20-27) | 22(18-26) | 0.002 |
| GCS,M(IQR) |  | 3(3-6) | 3(3-6) | 3(3-7) | 0.061 |
| NRS2002,M(IQR |  | 3(3-4) | 3(3-5) | 3(3-4) | 0.03 |
| RASS,M(IQR) |  | -3 (-3~-3) | -3(-3~-3) | -3 (-3~-3) | 0.031 |
| NIHSS,M(IQR) |  | 14(10-18) | 14(10-19) | 14(10-18) | 0.507 |
| WBC(×10^9^/L),M(IQR) |  | 11.2(8.7-14.4) | 11.04(8.31-14.5) | 11.3(8.9-14.3) | 0.481 |
| Hb(g/L)M,(IQR) |  | 120(105-135) | 115(101-134) | 123(107-135) | 0.002 |
| Alb(g/L),M(IQR) |  | 35.3(31.9-38.8) | 34.9(31.3-38.4) | 35.5(32.1-39) | 0.084 |
| BUN(mmol/L),M(IQR) |  | 6.1(4.7-8.6) | 6.2(4.7-9.5) | 6(4.6-8.1) | 0.202 |
| PCT(ng/mL),M(IQR) |  | 0.17(0.05-0.6) | 0.2(0.05-0.7) | 0.1(0.05-0.6) | 0.244 |
| CRP(mg/L),M(IQR) |  | 33.2(8.4-88.3) | 42(9-92.2) | 30(8.3-83.5) | 0.173 |
| Na(mmol/L)M(IQR) |  | 141(138-144) | 141(138-144) | 141(138-144) | 0.364 |
| K(mmol/L),M(IQR) |  | 3.7(3.4-4.0) | 3.7(3.4-4.0) | 3.7(3.5-4.0) | 0.671 |

Abbreviations used in this study included：ICH,Intracerebral haemorrhage；CI,Cerebral infarction；P-GID，Prior gastrointestinal disease；GISurg，History of gastrointestinal surgery；CP,continuous pumping;IP, intermittent pumping ;GD, gravity drip ;EN,enteral nutrition；Room temp,Room temperature； APACHE II, Acute Physiology and Chronic Health Evaluation II, range 0–71；GCS, Glasgow Coma Scale, range 3-15；NRS2002, Nutritional Risk Screening 2002, range 0-7；RASS, Richmond Agitation-Sedation Scale, range -5 to 4；NIHSS, National Institutes of Health Stroke Scale, range 0-42;WBC, White Blood Cell；Hb, Hemoglobin；Alb, Serum Albumin；BUN, Blood Urea Nitrogen；PCT, Procalcitonin；CRP，C-reactive Protein； Binary variables (1=Yes, 2=No) include: history of gastrointestinal disease, history of digestive system surgery, diabetes, hypertension, PPI use, oral potassium supplementation, probiotic use, sedative use, opioid use, gastrointestinal motility agent use, antibiotic use.Gender is coded as 1 (Male) or 2 (Female). Marital status is coded as: 1=Single, 2=Married, 3=Divorced, 4=Widowed. EN-related variables include: tube type (Gastric tube, nasointestinal tube), administration method (continuous pumping, intermittent pumping, gravity drip, bolus injection), formula type (TPF, SP, intact protein formula, peptide formula, mixed feeding). Laboratory parameters include: WBC, Hb, Alb, BUN, PCT, CRP, Na, K; all measurements are within the standard range. Other continuous variables include: age (years), room temperature (℃), ICU length of stay (days), mechanical ventilation duration (days), duration of enteral nutrition (days).
