# Supplementary material for: An evaluation based on explainable machine learning: analysis of risk factors for enteral nutrition-related diarrhea in patients with severe stroke
Source: Front Nutr. 2026 Aug 14;13:1893280. doi: 10.3389/fnut.2026.1893280 (PMC13521873; doi:10.3389/fnut.2026.1893280)
Supplement: Supplementary file 3 [file Table_2.docx]

Supplemental table2. Comparison of model performance with different feature sets based on cross-validation

| Model | Features | AUC | CI Lower | CI Upper | Z | P | Significance |
| --- | --- | --- | --- | --- | --- | --- | --- |
| Full13 | 13 | 0.820 | 0.683 | 0.957 | NA | NA | Reference |
| Features10 | 10 | 0.807 | 0.726 | 0.889 | 0.522 | 0.602 | Not Significant |
| Features7 | 7 | 0.811 | 0.686 | 0.936 | 0.455 | 0.649 | Not Significant |
| Features4 | 4 | 0.806 | 0.688 | 0.924 | 0.628 | 0.530 | Not Significant |
